# Machine learning-based CT radiomics model to discriminate the primary and secondary intracranial hemorrhage

**DOI:** 10.1038/s41598-023-30678-w

**Published:** 2023-03-06

**Authors:** Jianbo Lyu, Zhaohui Xu, HaiYan Sun, Fangbing Zhai, Xiaofeng Qu

**Affiliations:** 1 Department of Radiology, The Second Hospital of Dalian Medical University, No. 467 Zhongshan Road, Shahekou District, Dalian, 116023 China; 2 Department of Hernia and Colorectal Surgery, The Second Hospital of Dalian Medical University, No. 467 Zhongshan Road, Shahekou District, Dalian, 116023 China

**Keywords:** Neurology, Neuroscience, Diseases of the nervous system

## Abstract

It is challenging to distinguish between primary and secondary intracranial hemorrhage (ICH) purely by imaging data, and the two forms of ICHs are treated differently. This study aims to evaluate the potential of CT-based machine learning to identify the etiology of ICHs and compare the effectiveness of two regions of interest (ROI) sketching methods. A total of 1702 radiomic features were extracted from the CT brain images of 238 patients with acute ICH. We used the Select K Best method, least absolute shrinkage, and selection operator logistic regression to select the most discriminable features with a support vector machine to build a classifier model. Then, a ten-fold cross-validation strategy was employed to evaluate the performance of the classifier. From all quantitative CT-based imaging features obtained by two sketch methods, eighteen features were selected respectively. The radiomics model outperformed radiologists in distinguishing between primary and secondary ICH in both the volume of interest and the three-layer ROI sketches. As a result, a machine learning-based CT radiomics model can improve the accuracy of identifying primary and secondary ICH. A three-layer ROI sketch can identify primary versus secondary ICH based on the CT radiomics method.

## Introduction

Intracranial hemorrhage (ICH) is the most devastating type of stroke, with a reported 30-day mortality rate of nearly 40%, and it results in only one in five survivors living independently at six months after onset^1,2^. There are various risk factors and causes of ICH, and the classification of primary and secondary ICHs is now more widely accepted. The most common causes of primary ICH are hypertensive atherosclerosis and cerebral amyloid angiopathy, which account for approximately 42–80%^3^. Secondary ICH occurs in a minority of patients and is associated with coagulopathy, brain tumors, aneurysms, vascular anomalies, and thrombolytic treatment of ischemic stroke^4^. Surgical hematoma removal and conservative therapy are the main treatments for primary ICH^5^, but secondary ICH requires further attention to treat the more deep-rooted causes of the underlying disease. Therefore, the early identification of ICH etiology can significantly help guide the appropriate treatment strategy and optimize the prognosis of individual patients^6^.

Computed tomography (CT) imaging is the most used screening method for investigating suspected ICH^7^. However, follow-up imaging is required for the final diagnosis^6,7^. Therefore, some studies have investigated magnetic susceptibility weighted imaging or the^99m^Tc-MIBI SPECT techniques for ICH diagnosis, but the accuracy rates have reached only 68.83% and 65.5%, respectively. Other imaging approaches in the clinic have involved CT angiography (CTA), contrast-enhanced CT, or contrast-enhanced magnetic resonance imaging (MRI)^8^, but their diagnostic accuracy has been reported as only around 43%^9^. The imaging-based diagnosis of secondary ICH can further complicate clinical procedures because the enhanced signal characteristics of these lesions can be obscured by high signal attenuation or various hematoma-based T_1_-weighted MRI signal heterogeneities^10^.

Imaging technology is gradually developing toward automated analysis and high-throughput extraction of quantitative features. Nevertheless, semi-supervised approaches using human input have allowed this avenue to arrive at the conception of the field of radiomics^11,12^. Along the same lines, machine learning is a field of computer science that uses statistical techniques to enable computers to “learn” patterns within large datasets without being explicitly programmed. In past years, machine learning algorithms have been applied to cancer research to predict genotype or patient prognosis pre-operatively based on radiomics features^13,14^.

To the best of our knowledge, the current work is the first instance in which radiomics and machine learning have been employed for the differential diagnosis of primary and secondary ICH. This study used a radiomics approach to identify features in CT images of patients with ICH of unknown etiology^15,16^ and to evaluate their predictive performance for the disease type. We further evaluated radiomics-based machine learning models using features from two ROI sketching approaches to evaluate the role of feature extraction on differential ICH prediction. The results of this machine learning approach were compared with those of routine visual assessment performed by two experienced radiologists.

## Materials and methods

### Patients and data management

**Figure 1:**
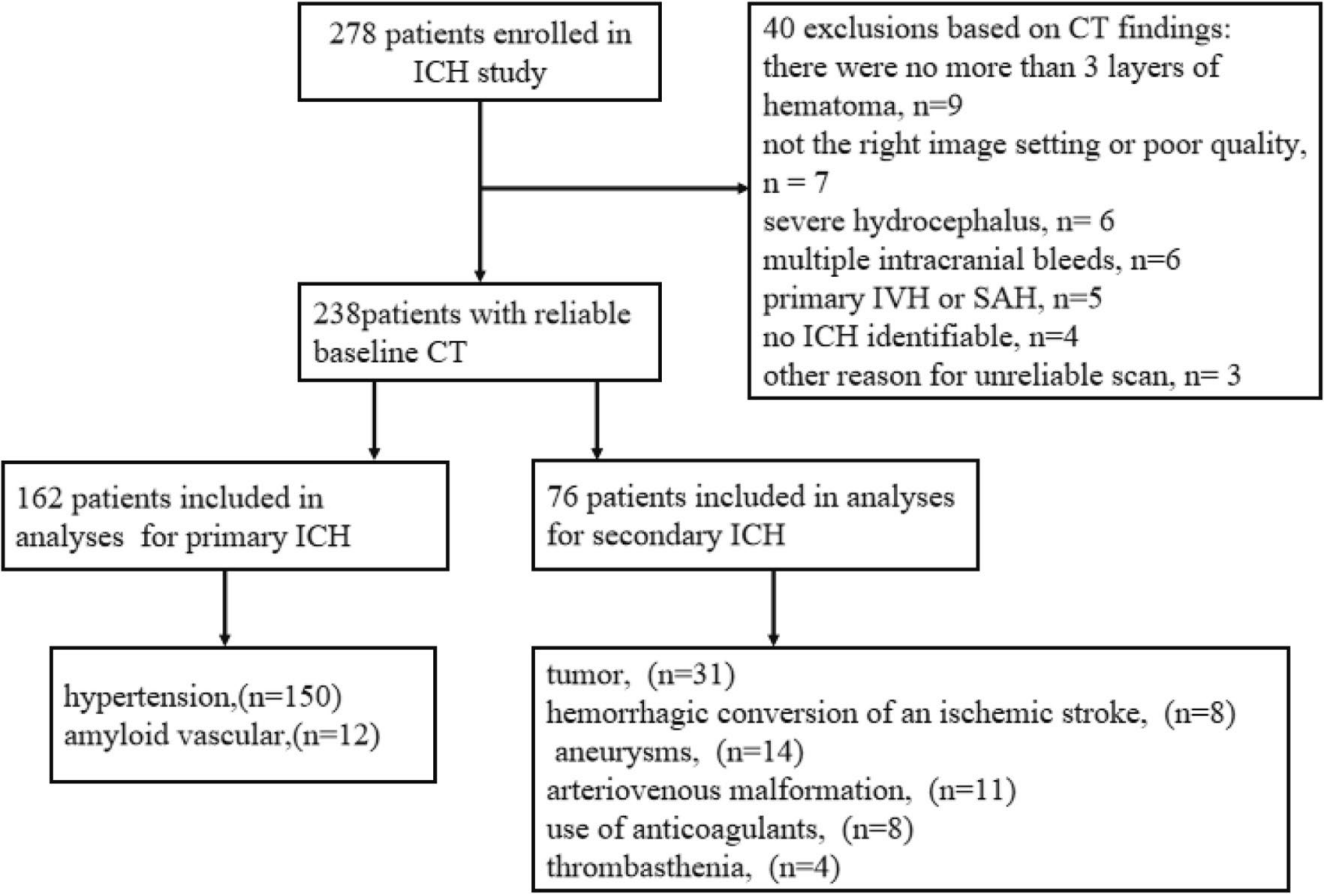
Patient inclusion/exclusion criteria.

This study systematically reviewed the clinical database of CT images from 238 patients with acute ICH from January 2019 to January 2021 at our hospital. The patient inclusion criteria were as follows: (1) acute, non-traumatic ICH; (2) completion of cranial CT within 24 h; (3) imaging follow-up (complete resorption of the hematoma shown on CT images), clinical or pathological confirmation of the etiology of acute ICH. The patient exclusion criteria were as follows: (1) intraventricular hemorrhage or subarachnoid hemorrhage predominant; (2) multiple hemorrhagic lesions; (3) incorrect image setting or poor imaging quality; (4) unknown etiology; (5) hematoma present at ≤ 3 layers; (6) The hematoma selected at the three largest layers is influenced by surrounding tissues (such as the skull). Patients with ICH were followed up for regular CT examinations until complete resorption of the hematoma was observed, and no other lesions were found. We classified this category as primary ICH and included such cases in this study. The study population enrollment process and the etiology of the patients in the secondary ICH group are shown in Fig. 1.

### CT data acquisition

All patients underwent CT scans in the same order on a 128 biplane scanner (Philips 128-layer micro flat CT). Scans were performed from the cranial vault to the base of the skull (120 kV, 210–439 mA, 5.0 mm slice thickness), and quality checks were performed to exclude severe motion artifacts.

### Lesion segmentation

The images were aligned and resampled using the 3D Slicer Version 2.6 software (www.slicer.org) to unify the resolution of the CT images^17^. When drawing regions of interest (ROI) on the CT image, the color scale was thresholded with a lower bound of 25–40 HU and an upper bound of 130 HU. When an ICH was located adjacent to the skull, skull stripping was performed before drawing the ROI by masking pixels higher than 130 HU^6^. We used the 3D Slicer software to draw an ROI of the hematoma slice-by-slice on the CT image stack. The ROI aimed to sketch the edge of the hematoma and avoid the surrounding edema. The VOI is to sketch the whole of the hematoma (from the first layer where the hematoma appears until the last layer where the hematoma disappears) to obtain the area of interest of the overall volume with the same method to sketch three layers ROI of the hematoma. The three layers in this study are those selected from each patient's axial CT image showing the largest area of the hematoma and the two layers above and below it. Segmentation was performed by two radiologists with five years of experience. Each segmentation was reviewed by a senior radiologist and technical engineer with ten years of experience.

### Feature extraction

Image sketching for feature extraction was performed using 3D Slicer for medical image computation and visualization^17^. Image features, such as shape, first-order statistics, and texture features, were extracted from the VOI and three-layer ROI sketching images.

### Feature selection

The feature selection was performed using the Darwin research platform (Yizhun Medical AI Co. Ltd). For classification, all cases were randomly divided into training and test sets comprising 70% and 30% of the data, respectively^18^. We did feature selection building on the training set, and evaluated on the test set, it wasdivided into two steps. The first step used Select K Best univariate feature selection method. In this step, we setedthe parameter K to 140 based on experience, and select the f_calssif function to extract the feature. The second step used the least absolute shrinkage and selection operator (LASSO).

### Machine learning

**Figure 2:**
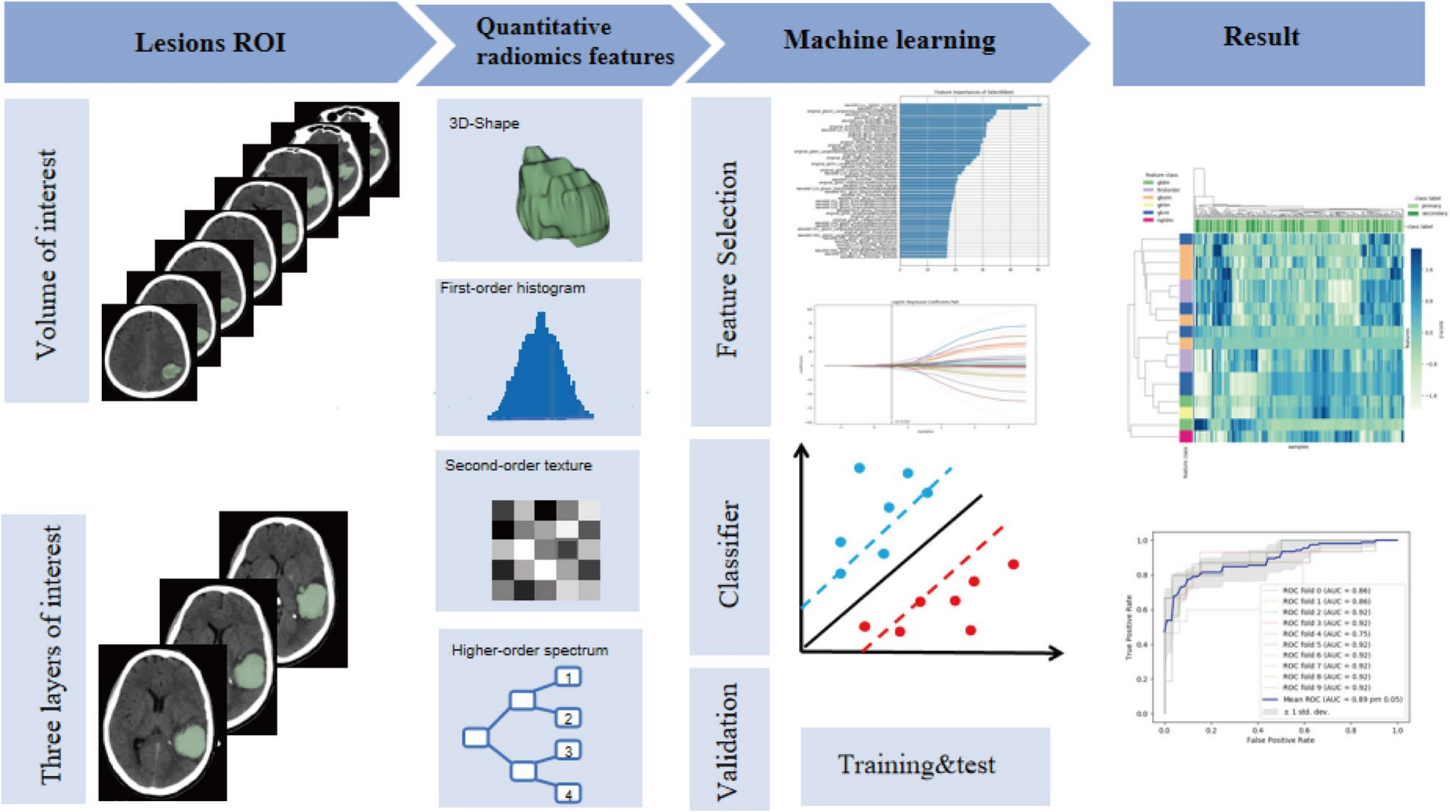
Workflow of image post-processing.

The proposed radiomics analysis was performed using the Darwin research platform. The extracted features were also preprocessed and normalized between 0 and 1. Classification of features was using support vector machines (SVM). The training data was then divided into ten subsamples using ten-fold cross-validation, and the data collected from each subsample was retained to validate the model while the data from the remaining nine subsamples were trained. Each subsample was cross-validated, and an average of ten cross-validations was taken to assess the generalization of the classification models and the accuracy of the algorithm^19^. The mean area under the curve (AUC) of the receiver operator characteristic (ROC) curve, sensitivity, specificity, and accuracy were used to evaluate the diagnostic efficacy of the model. A graphical flow chart of this CT-based machine learning approach to predict the etiology of ICH is shown in Fig. 2.

### Radiologist evaluation

As a clinical standard and control, two radiologists with more than ten years of experience in central neurological diagnosis also predicted the category of ICH based on their reading of the CT images. Each ICH image was categorized as “primary” or “secondary.” Physicians did not know the actual results or each other’s predictions.

### Statistical analyses

The SPSS software (version 26.0; IBM Corporation, Armonk, NY, United States) was used to perform all statistical analyses. Differences between groups were assessed using a *t*-test, a Mann–Whitney U test, or a χ^2^-test. We performed a binary logistic regression analysis to identify covariates associated with identifying the etiology of ICH. Odds ratios (OR), 95% confidence intervals (CI), and *P* values were presented for all selected variables, with *P* < 0.05 indicating statistical significance. ROC curves were used to predict the sensitivity and specificity of primary versus secondary ICH. The concordance of diagnostic results between the two radiologists was determined by evaluating the intraclass correlation coefficient (ICC). ROC curves were plotted against physicians' diagnoses using MedCalc for Windows (version 15.0, MedCalc Software, Ostend, Belgium).

### Ethics approval and consent to participate

This retrospective study was approved by the ethics review board of Second Hospital of Dalian Medical University. The requirement for informed consent was waived by our Review Board owing to the retrospective nature of the current study. The methods in the current study were performed in accordance with the relevant guidelines and regulations.

## Results

### Study population

A total of 238 patients were included in this study for the final analysis. Two hundred and thirty-eight patients were admitted within 72 h of ICH onset and underwent follow-up CT scans. Of these patients, 162 were diagnosed with primary ICH either by clinical, pathological, or follow-up CT results, with 76 being diagnosed with secondary ICH. In the primary ICH group, hypertension (n = 150) and amyloid vascular (n = 12) were the underlying causes of the disease. By contrast, in the secondary ICH group, tumor (n = 31), hemorrhagic conversion of an ischemic stroke (n = 8), aneurysms (n = 14), arteriovenous malformation (n = 11), use of anticoagulants (n = 8), and thrombasthenia (n = 4) were the underlying causes. There was a statistical difference in gender (*P* = 0.003), the time from onset to first CT examination (*P* = 0.003), hematoma location (*P* = 0.003), hyperlipidemia (*P* = 0.040), burst into the ventricle (*P* = 0.029), and hypertension (*P* ≤ 0.01) between the primary and secondary ICH groups (Table 1). Binary logistic regression analysis showed that gender (*P* = 0.005), hematoma location (*P* ≤ 0.01), onset to examination time (*P* = 0.002), and hypertension (*P* = 0.002) were independent factors for identifying primary versus secondary ICH (Table 2).

**Table 1:** Demographic information of the study population.

Baseline characteristics	Primary ICH (n = 162)	Secondary ICH (n = 76)	*P*-value
Age (years), mean (mean ± SD)	58.12 ± 13.9	58.20 ± 15.6	0.971
Sex male, n (%)	122(75.3)	43(56.5)	0.003
Time onset to imaging (h), median (IQR)	5(3–13.25)	15(4.25–72)	0.000
Localization n (%)			0.000
Lobar	34(21.0)	47(61.2)	
Basal ganglia	94(58.0)	21(27.6)	
Cerebellum	12(7.4)	5(6.6)	
Thalamus	22(13.6)	3(4.0)	
With hyperlipidemia, n (%)	37(22.8)	27(35.5)	0.040
Burst into ventricle, n (%)	27(16.67)	22(28.9)	0.029
With hypertension on admission, n (%)	118(72.8)	34(44.7)	0.000

Continuous variables are represented as mean ± standard deviation (SD) and categorical variables as number (n), and percentages (%). IQR=inter-quartile range; ICH=intracranial hemorrhage; HU=hounsfield unit.

**Table 2:** Binary logistic regression modeling and the prediction of favorable outcome.

	Odds ratio, Exp (B)	95% CI	*P* value
Gender			
Male	Reference*		
Female	2.84	1.36–5.92	0.005
Onset to examination time	1.00	0.97–1.02	0.002
Localization			0.000
Basal ganglia	Reference*		
Lobar	4.82	2.23–10.22	0.000
Cerebellum	1.45	0.37–5.67	0.597
Thalamus	0.52	0.12–2.19	0.37
With hypertension on admission	0.34	0.17–0.68	0.002

Binary analysis was used with a stepwise forward selection. All selected variables are presented with OR, 95%CI, and *P* value.

*Reference point for other OR calculations.

### Radiomics feature selection

**Figure 3:**
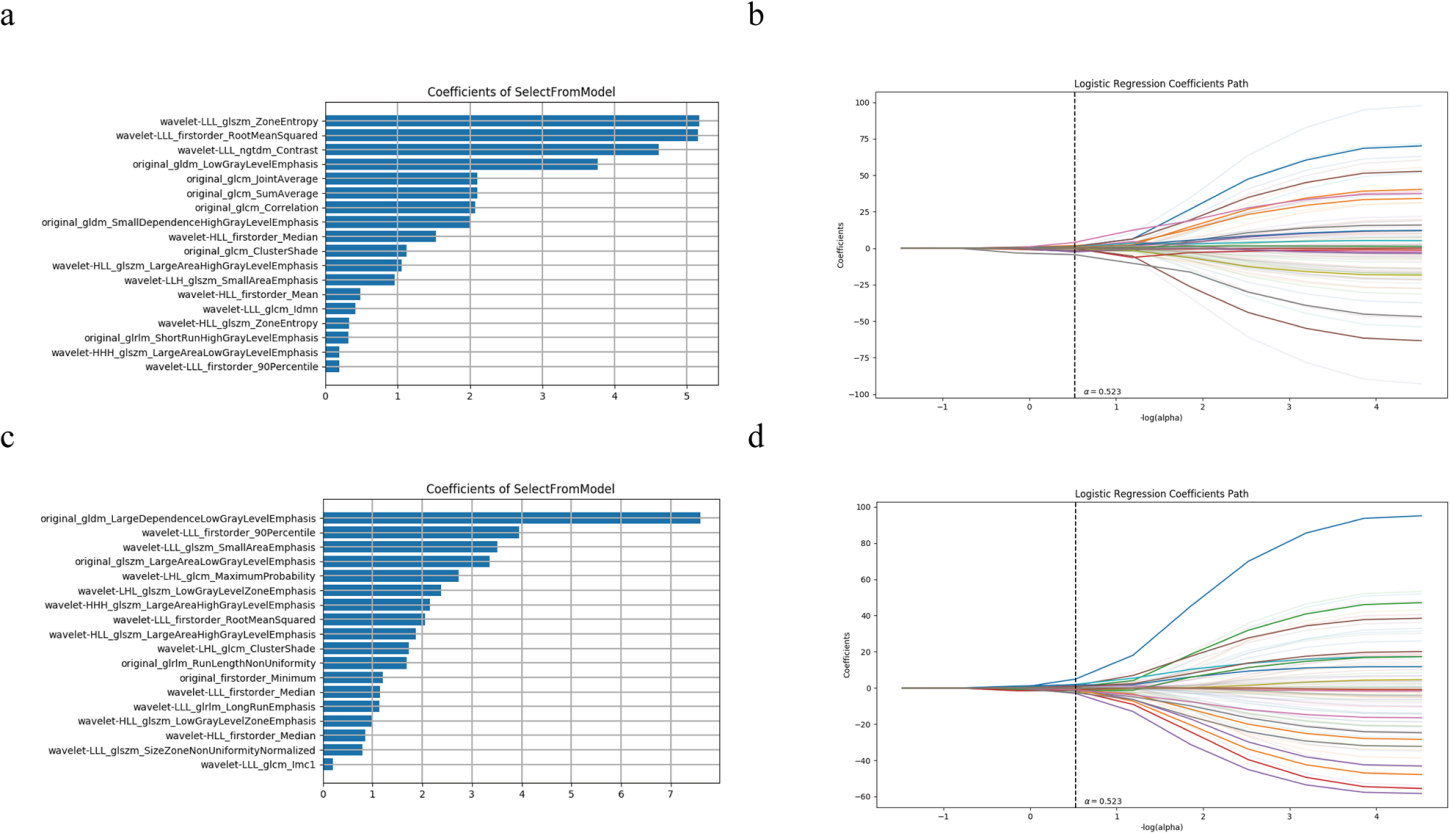
Feature selection using LASSO logistic regression. (**a**) The coefficients of the remaining eighteen features in the VOI sketching method. (**b**) The regression coefficients of LASSO in the VOI sketching method. (**c**) The coefficients of the remaining eighteen features in the three-layer sketching method. (**d**) The regression coefficients of LASSO in the three-layer sketching method.

**Figure 4:**
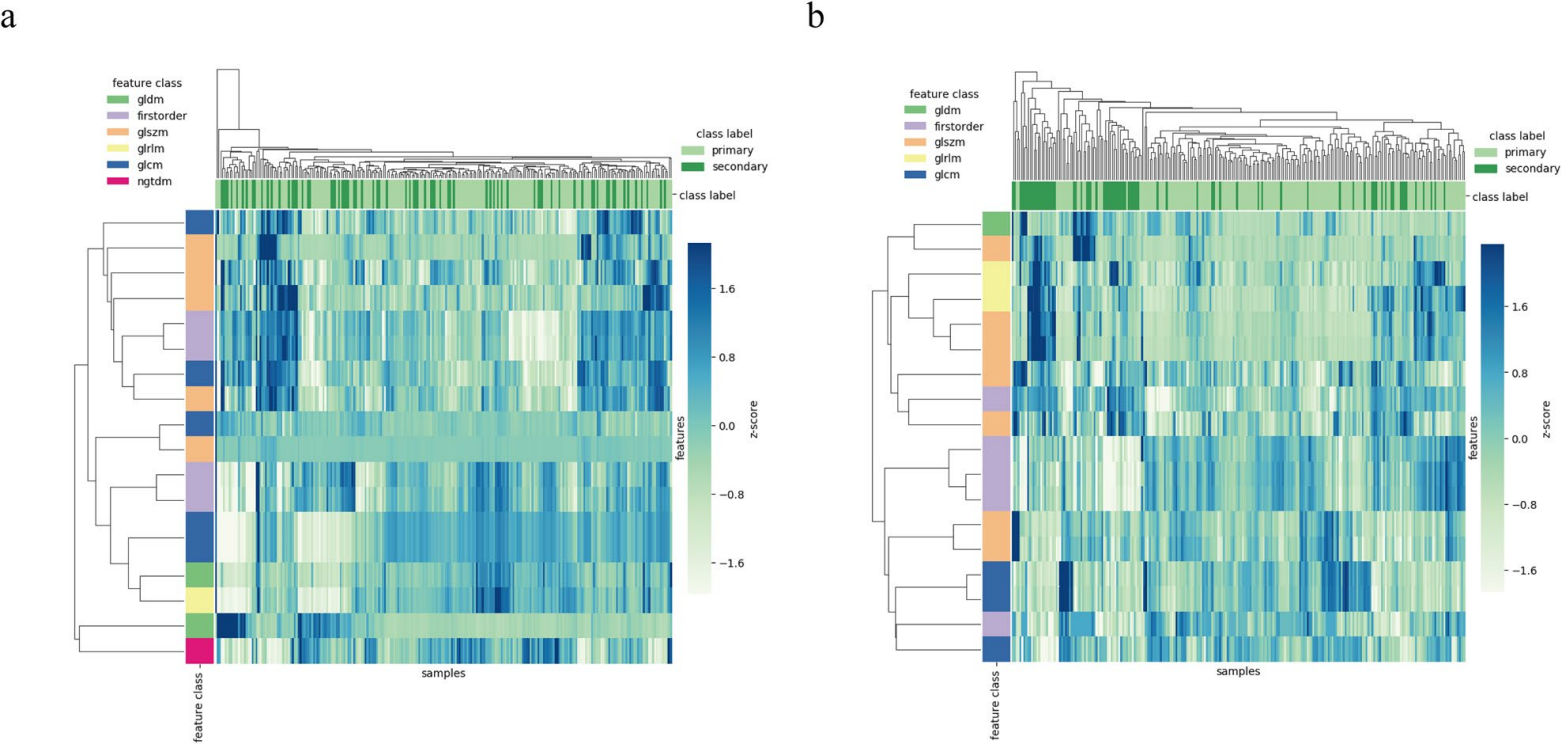
Heat map of the selected radiomics features using LASSO logistic regression in the VOI (**a**) and three-layer (**b**) sketching methods.

A total of 1702 quantitative image features were extracted from the VOI and three-layer ROI sketching images (n = 851 per method). These features included 36 first-order features, 150 s-order features, 1488 wavelet features, and 28 shape features. The final 18 best features in the VOI sketching images were extracted, where the wavelet-LLL_GLSZM_ZoneEntropy feature exhibited the largest feature weight (Fig. 3a, b and Table 3). Similarly, the 18 best features were extracted in the three-layer ROI sketching images, among them the original_GDLM_LargeDependenceLowGrayLevelEmphasis feature exhibited the largest feature weight (Fig. 3c, d and Table 4). The features heat maps in Fig. 4 show that there are remarkable differences in the clustering of the primary and secondary ICH classes for the VOI and the three-layer ROI sketch.

**Table 3:** Target image histology features and their feature classes and filters based on the SVM classifier model with the VOI sketching method.

Radiomic feature	Radiomic class	Filter
Zone entropy	glszm	Wavelet-LLL
Root mean squared	first order	Wavelet-LLL
Contrast	ngtdm	Wavelet-LLL
Low gray level emphasis	gldm	Original
Joint average	glcm	Original
Sum average	glcm	Original
Correlation	glcm	Original
Small dependence high gray level emphasis	gldm	Original
Median	first order	Wavelet-HLL
Cluster shade	glcm	Original
Large area high gray leve emphasis	glszm	Wavelet-HLL
Small area emphasis	glszm	Wavelet-LLH
Mean	first order	Wavelet-HLL
Idmn	glcm	Wavelet-LLL
Zone entropy	glszm	Wavelet-HLL
Short run high gray level emphasis	glrlm	Original
Large area low gray level emphasis	glszm	Wavelet-HHH
90 percentile	first order	Wavelet-LLL

GLSZM = Gray-Level Size Zone Matrix; GLRLM = Gray-Level Run Length Matrix; GLCM = Gray-Level Co-occurrence Matrix; GLDM = Gray Level Dependence Matrix; NGTDM = Neighbouring Gray Tone Difference Matrix; VOI = volume of interest.

**Table 4:** Target image histology features and their feature classes and filters based on the SVM classifier model with the three-layer sketching method.

Radiomic feature	Radiomic class	Filter
Large dependence low gray level emphasis	gldm	Original
90 percentile	first order	Wavelet-LLL
Small area emphasis	glszm	Wavelet-LLL
Large area low gray level emphasis	glszm	Original
Maximum probability	glcm	Wavelet-LHL
Low gray level zone emphasis	glszm	Wavelet-LHL
Large area high gray level emphasis	glszm	Wavelet-HHH
Root mean squared	first order	Wavelet-LLL
Large area high gray level emphasis	glszm	Wavelet-HLL
Cluster shade	glcm	Wavelet-LHL
Run length non uniformity	glrlm	Original
Minimum	first order	Original
Median	first order	Wavelet-LLL
Long run emphasis	glrlm	Wavelet-LLL
Low gray level zone emphasis	glszm	Wavelet-HLL
Median	first order	Wavelet-HLL
Size zone non uniformity normalized	glszm	Wavelet-LLL
Imc1	glcm	Wavelet-LLL

GLDM = Gray Level Dependence Matrix; GLSZM = Gray-Level Size Zone Matrix; GLCM = Gray-Level Co-occurrence Matrix; GLRLM = Gray-Level Run Length Matrix.

### Classifier performance

**Figure 5:**
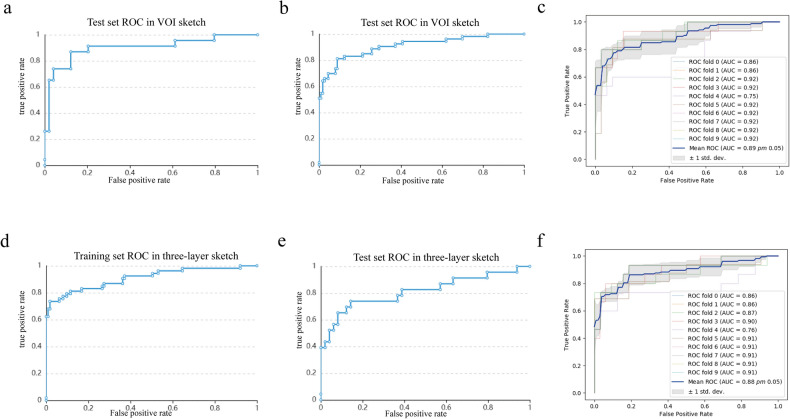
ROC curves for the differential diagnosis of primary and secondary ICH based on an SVM classifier. ROC curves for the VOI sketching method using the training (**a**) and test (**b**) sets. ROC curves for the three-layer sketching method using the training (**c**) and test (**d**) sets. ROC curves of the SVM-based classification with ten-fold cross-validation for the VOI (**e**) and three-layer (**f**) sketching methods.

The AUC value of the VOI sketch in the training set was 0.91 (95% CI: [0.84, 0.98], and the test set was 0.90 (95% CI: [0.80, 1]) (Fig. 5a, b, Table 5). In addition, the mean AUC value of the ten-fold cross-validation was 0.89 ± 0.05 (Fig. 5c, Table 6). For the three-layer ROI sketch, the AUC value of the training set was 0.91 (95% CI: [0.83, 0.99], and in the test group, it was 0.81 (95% CI: [0.66, 0.98]) (Fig. 5d–e, Table 5). Furthermore, the mean AUC value of ten-fold cross-validation was 0.88 ± 0.05 (Fig. 5f, Table 6).

**Table 5:** AUC, sensitivity, specificity, and overall accuracy of SVM prediction models for the training and test sets for the two sketching methods.

Sketch method	Training set (n = 166)				Test set (n = 72)			
	AUC [95%CI]	Sensitivity	Specificity	Accuracy	AUC [95%CI]	Sensitivity	Specificity	Accuracy
VOI sketching images	0.91 [0.84,0.98]	0.98	0.64	0.87	0.90 [0.80,1]	0.95	0.65	0.86
Three-layer sketching images	0.91 [0.83,0.99]	0.98	0.74	0.90	0.81 [0.66,0.98]	0.92	0.61	0.82

AUC=area under the receiver operating characteristic curve.

**Table 6:** Efficacy and stability of SVM classifier for predicting the differential diagnosis of primary and secondary ICH based on two sketching methods.

Sketch method	Classification	Mean AUC	Precision	Recall	Sensitivity	Specificity	F1-score
VOI sketching images	Primary ICH	0.89 ± 0.05	0.85	0.96	0.96	0.65	0.90
	Secondary ICH	0.89 ± 0.05	0.88	0.65	0.65	0.96	0.75
Three-layer sketching images	Primary ICH	0.88 ± 0.05	0.83	0.92	0.92	0.61	0.87
	Secondary ICH	0.88 ± 0.05	0.78	0.61	0.61	0.92	0.68

### Radiologist evaluation

**Figure 6:**
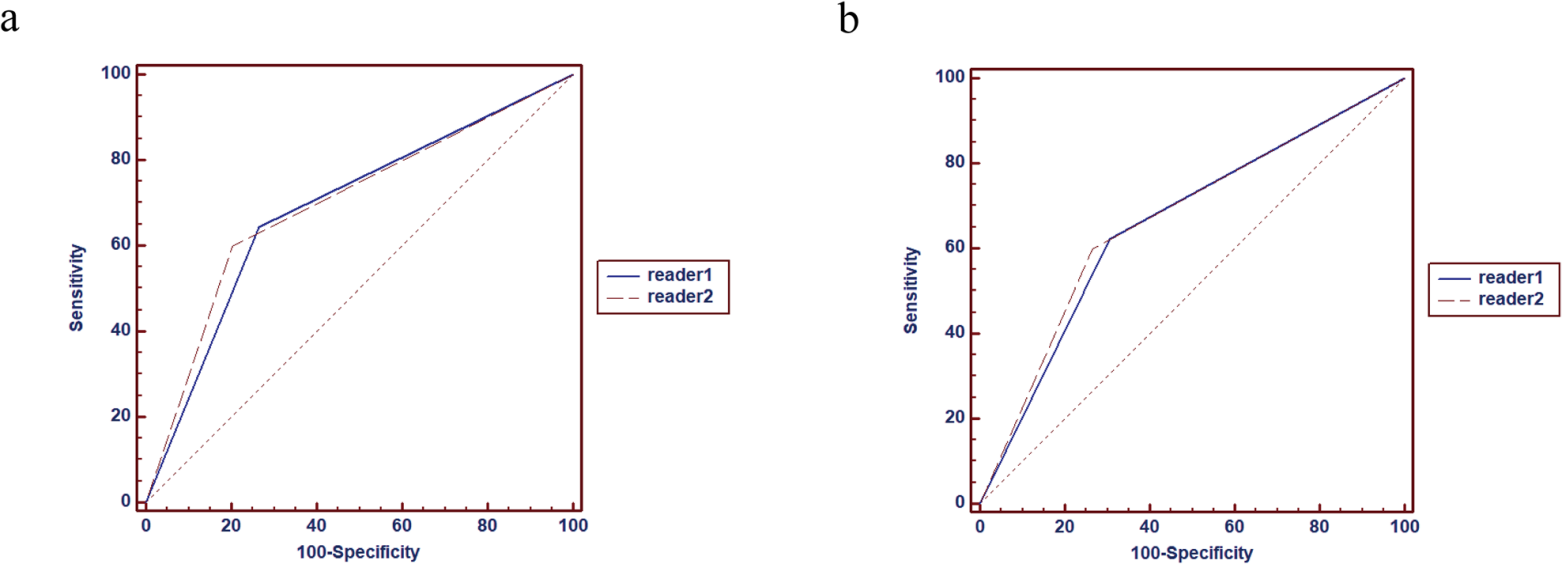
ROC curves for the differential diagnosis of primary and secondary ICH based on radiologists’ reading. The classification results of human readers 1 and 2 for the VOI (**a**) and three-layer (**b**) sketching methods.

For the VOI sketch, the AUC value for the two radiologists to identify the type of ICH was 0.69 (95% CI: [0.59, 0.78]) and 0.70 (95% CI: [0.60, 0.79]), respectively. The ICC result of two radiologists diagnosing the etiology of ICHs was 0.90 (95% CI: [0.88, 0.92]) (Fig. 6a, Table 7). By contrast, the AUC values for the two radiologists with the three-layer ROI sketch were 0.66 (95% CI: [0.55, 0.75]) and 0.67 (95% CI: [0.56, 0.76]), respectively. The ICC result of two radiologists diagnosing the etiology of ICHs was 0.86 (95% CI: [0.83, 0.90]) (Fig. 6b, Table 7).

**Table 7:** Efficacy and stability of physician prediction of the differential diagnosis of primary and secondary ICH based on the two sketching methods.

Sketch method	Radiologists' predictions	AUC	95%CI	Precision	Recall	Sensitivity	Specificity	ICC	95%CI
VOI sketching images	Reader 1	0.69	0.59–0.78	0.68	0.64	0.64	0.73	0.90	0.88–0.92
	Reader 2	0.70	0.60–0.79	0.72	0.60	0.60	0.80		
Three-layer sketching images	Reader 1	0.66	0.55–0.75	0.65	0.62	0.62	0.69	0.86	0.83–0.90
	Reader 2	0.67	0.56–0.76	0.69	0.60	0.60	0.73		

ICC=intraclass correlation coefficient.

## Discussion

### Efficacy of CT signs and clinical indicators to identify primary and secondary ICHs

This study demonstrated that various clinical indicators and imaging characteristics can be used to distinguish primary from secondary ICH and that a CT-based radiomics classifier performs better than subjective physician readings. We found that gender, the time from onset to first CT examination, hematoma location, hyperlipidemia, burst into the ventricle, and hypertension were significant predictors for distinguishing primary and secondary ICHs (all *P* < 0.05). Furthermore, the binary logistic regression analysis showed that female patients with lesions in the lobes were more likely to suffer from secondary ICH. However, patients with hypertension at admission were more likely to suffer from primary ICH. Previous studies have also shown that ICHs caused by cavernous hemangioma are more likely to occur in young women^20^.

The location of the secondary ICH largely depends on that of the primary lesion. The results of the present study suggest that primary ICH usually occurs in deep brain structures, and secondary ICH favors the cerebral lobes. These results are consistent with the findings of Dastur et al.^21^, indicating that ICH caused by hypertension was mainly located in the basal ganglia, while ICH caused by anticoagulants, thrombocytopenia, metastatic tumors, cerebrovascular malformations, or cerebral aneurysms was mostly located in the cerebral lobes. However, not all ICHs in the basal ganglia are primary, and not all cerebral lobar hemorrhages are secondary. Nevertheless, these convincing characteristics provide clinicians with important biomarkers for diagnosing ICH (Tables 1 and 2).

### Efficacy of radiomics in distinguishing primary from secondary ICHs

For ICH of an unclear nature, CTA, DSA, contrast-enhanced CT or MRI, and MRA are generally used to further determine the etiology^8,22^, with CTA being the most frequently used imaging technique^23^. Along these lines, Alshumrani et al. found that although 68% of CT scans showed hemorrhage, nearly 58% of CTA examinations were normal or did not show associated abnormalities. This suggests that in most cases, CTA cannot determine the cause of the bleeding^9^. Therefore, radiomics models can significantly improve the diagnosis and help in imaging-based investigations and treatment strategies. For example, Choi et al.^6^ aimed to investigate the diagnostic value of CT densitometry for neoplastic and non-neoplastic etiologies of acute ICH. The ROC curve showed that the 5th and 25th percentile values had the highest diagnostic performance. Nawabi et al.^24^ further assessed the potential of machine learning-based prediction for identifying acute ICH etiology, which resulted in a prediction AUC of 0.89 and specificity and sensitivity values of greater than 80%. Unlike these previous studies, we divided the etiology of ICHs into primary and secondary, not limited to tumorigenic and non-tumorigenic etiologies, and utilized both VOI and three-layer ROI sketches to predict primary versus secondary ICHs. We found that the VOI sketch achieved an AUC value of 0.90 (95% CI: [0.80, 1]) (mean AUC value 0.89 ± 0.05 of ten-fold cross-validation) and that the three-layer ROI sketch achieved an AUC value of 0.81 (95% CI: [0.66, 0.99]) (mean AUC value 0.88 ± 0.05 of ten-fold cross-validation). Overall, we concluded that these machine learning algorithms produced more accurate results on all assessment metrics compared to radiologists’ predictions.

In the current work, we used an SVM classifier to train prediction models for the etiological discrimination of ICH. SVM is a widely used method for processing many images and is based on a kernel approach to finding an optimal hyperplane in n-dimensional space that best separates the feature set into their respective classes^25^. SVM uses specific kernel methods for feature transformation, and the choice of kernel functions and other factors may affect the performance of SVM models. However, there is no standard to measure which kernel function is the best regarding recognition effectiveness. The selection of the best kernel function needs to be based on the nature of the problem and then implemented through extensive experimentation. Data transformed by kernel functions can be partitioned more easily, a feature that improves model stability and avoids overfitting during training^25^. This study selected the kernel function Radial Basis Function. SVM balances the variance and bias of the input data and is, therefore, best suited for studies with a small number of measurements. Other classification models, such as logistic regression and decision trees, operate in the original feature space, which lacks flexibility and may not always achieve a high accuracy^26^.

Segmentation methods include manual, semiautomatic, and automatic approaches^27^. We used two manual sketching methods in this study and compared their predictive values to identify the cause of ICH. The VOI sketch contained information from the lesion in 3-dimensional space, providing more quantitative features regarding ICH subtype identification^28^. However, the three-layer ROI sketch only included information obtained from three specific planes that included the lesion. The results of the study showed that the AUC values obtained by both sketching methods were in the range of 0.7 < AUC ≤ 0.9, which is a level of moderate diagnostic value. This similarity may be because small portions of the skull were included when the hematoma VOI was sketching. Such a process may have led to a less precise segmentation near the skull, which could lead to misleading interpretations of such features^29^. Although we set an upper threshold of 130 HU to avoid the influence of the skull on the lesion ROI, it was much easier to avoid the inclusion of the skull using the three-layer ROI sketch. In addition, the three layers may include most of the important information about the hematoma. Therefore, the three-layer ROI sketch can be used as a simple and rapid radiomics image segmentation method for the differential diagnosis of primary versus secondary ICH. However, the three-layer ROI sketch needs further investigation to confirm its true value and is expected to be useful in future radiomics studies.

### Correlation of radiomics features with lesions

CT image texture analysis is a potential biomarker for assessing and quantifying tumor heterogeneity. Although there may be heterogeneity in images associated with scanning techniques (e.g., image noise and artifacts)^30^, the texture features of CT images are highly consistent, so CT-based radiomics analysis is feasible^31–33^. Many studies have suggested that CT-based textural features may be related to the characteristics of histopathology-related lesions, with spatial heterogeneity observed in cellular density, angiogenesis, necrosis, and microstructural changes^34–37^. In terms of image characteristics, we commonly use gray contrast, depth, uniformity, and roughness of texture as basic features to identify lesion and non-lesion images^38^. In this study, 18 target features were obtained using two sketching methods after two dimensionality reduction processes. These higher-order features can explain the different properties and spectral components of the ROI and can quantify the heterogeneity of the image^39,40^. The grayscale area size matrix (GLSZM) accounted for the largest proportion of all target features (12/36,33.3%). GLSZM quantifies the grayscale regions in an image, which are defined as a certain number of connected voxels sharing the same grayscale intensity. This feature shows that the uniformity of the texture is related to the size of the gray area^41^. Furthermore, any change in the size of a GLSZM can sharply reflect the difference between primary and secondary ICH. Therefore, GLSZM plays an important role in identifying the etiology of ICH.

The weight of the wavelet-LLL_GLSZM_ZoneEntroy was the largest among the features extracted from the VOI sketch. It measures the uncertainty or randomness in the distribution of zone sizes and grayscale values, with a higher value indicating more heterogeneity in the texture patterns. This feature indicates a high variability of zone sizes and grayscale values in primary versus secondary ICH images, confirming that structural features may be associated with the heterogeneity of lesions. By contrast, the weight of original_GLDM_LargeDependenceLowGrayLevelEmphasis was the largest among the features extracted from the three-layer ROI sketch. The gray-level dependence matrix (GLDM) mainly quantifies the gray dependence in the image, which is defined as the number of connected voxels within a specific distance and is determined by the central voxel^26^. The LargeDependenceLowGrayLevelEmphasis feature measures the joint distribution of dependence with lower grayscale values. The large weight of the GLDM feature indicates that ICH etiology can be better predicted using low grayscale values extracted using the three-layer ROI sketch and indicates that greater heterogeneity exists between primary and secondary ICH. The above results may be because the secondary ICH has a wide classification of etiologies, and the hematoma components due to different etiologies may not be identical, leading to differences between lesions on CT images. Such differences are usually not identifiable to the human eye. However, future studies require further investigation into how these radiomics features correlate with primary and secondary ICH.

Our study has various limitations. First, our study was a retrospective analysis with small sample size and used a single machine scan, which still needs to be validated by a larger prospective multicenter study. Second, the types of secondary ICH etiologies we included were limited, and the differences between ICH from different etiologies are unclear. Finally, the ROIs in this study were artificially defined, which implied a certain degree of supervision in the machine learning process. This concept was further complicated by the lack of strict reference standards for selecting the three layers. In the future, optimized image plane selection would help drive this approach towards purely unsupervised approaches that would be ideal for clinical integration.

## Conclusion

The CT radiomics model based on machine learning established in this study effectively discriminated between primary and secondary ICH. The VOI sketch exhibited the best performance in discriminating primary from secondary ICH. However, the three-layer ROI sketch produced highly similar results. Overall, a radiomic approach based on CT is expected to provide an imaging basis for clinicians to evaluate the prognosis of ICH and formulate personalized treatment plans.

## Data Availability

The datasets used and/or analyzed during the current study are available from the corresponding author upon reasonable request.
